# Nationwide carrier detection and molecular characterization of β-thalassemia and hemoglobin E variants in Bangladeshi population

**DOI:** 10.1186/s13023-020-1294-z

**Published:** 2020-01-15

**Authors:** Farjana Akther Noor, Nusrat Sultana, Golam Sarower Bhuyan, Md Tarikul Islam, Mohabbat Hossain, Suprovath Kumar Sarker, Khaleda Islam, Waqar Ahmed Khan, Mujahida Rahman, Syeda Kashfi Qadri, Hossain Uddin Shekhar, Firdausi Qadri, Syed Saleheen Qadri, Kaiissar Mannoor

**Affiliations:** 1 Laboratory of Genetics and Genomics, Institute for Developing Science and Health Initiatives, Mohakhali, Dhaka, 1212 Bangladesh; 2 Department of Biochemistry and Molecular Biology, University of Dhaka, Shahbagh, Dhaka, 1000 Bangladesh; 3 Department of Virology, Dhaka Medical College Hospital, Shahbagh, Dhaka, 1000 Bangladesh; 4 Directorate General of Health Services, MoHFW, Government of Bangladesh, Mohakhali, Dhaka, 1212 Bangladesh; 5 Department of Biochemistry and Molecular Biology, Dhaka Shishu Hospital, Sher-e-Bangla Nagar, Dhaka, 1207 Bangladesh; 6 Department of Hematology, Bangabandhu Sheikh Mujib Medical University, Shahbagh, Dhaka, 1000 Bangladesh; 7 Department of Pediatric Medicine, KK Women’s and Children’s Hospital, 100 Bukit Timah Road, Singapore, Singapore; 8 Department of Enteric and Respiratory Infectious Diseases, Infectious Diseases Division, International Centre for Diarrhoeal Disease Research, Mohakhali, Dhaka, 1212 Bangladesh

**Keywords:** ß-thalassemia, Carrier frequency, HRM curve analysis, Detection accuracy, Novel mutation, National policy

## Abstract

**Background:**

ß-thalassemia is one of the most common inherited blood disorders in the world and a major deterrent to the public health of Bangladesh. The management of thalassemia patients requires lifelong frequent blood transfusion and the available treatment options are unsatisfactory. A national policy on thalassemia prevention is mandatory in Bangladesh. However, precise and up-to-date information on the frequency of ß-thalassemia carriers are missing due to lack of accurate diagnostic approaches, limited access to information and absence of national screening program. This study aims to determine the nationwide carrier frequency of hemoglobin E (HbE) and β- thalassemia and mutation spectrum among the carriers using molecular, hematological and biochemical methods.

**Methods:**

The study enrolled a total of 1877 individuals (60.1% male and 39.9% female) aged between 18 and 35 years. Total sample size and its division-wise breakdown were calculated in proportion to national and division-wise population. Venous blood was collected and subjected to CBC analysis and Hb-electrophoresis for each participant. Serum ferritin was measured to detect coexistence of iron deficiency anemia with thalassemia carrier. DNA-based High Resolution Melting (HRM) curve analysis was performed for confirmation of carrier status by mutation detection.

**Results:**

Of 11.89% (95% CI, 10.43–13.35) carriers of β-globin gene mutations, 8.68% (95% CI, 7.41–9.95) had HbE trait (ETT) and 2.24% (95% CI, 1.57–2.91) had beta-thalassemia trait (BTT). Among eight divisions, Rangpur had the highest carrier frequency of 27.1% (ETT-25%, BTT-2.1%), whereas Khulna had the lowest frequency of 4.2% (ETT-4.2% only). Moreover, α- thalassemia, HbD trait, HbE disease, hereditary persistence of HbF were detected in 0.11, 0.16, 0.43 and 0.16% participants, respectively. HRM could identify two individuals with reported pathogenic mutations in both alleles who were erroneously interpreted as carriers by hematological indices. Finally, a total of nine different mutations including a novel mutation (c.151A > G) were detected in the β-globin gene.

**Conclusions:**

Since carrier frequency for both HbE and β-thalassemia is alarmingly high in Bangladesh, a nationwide awareness and prevention program should be made mandatory to halt the current deteriorating situations. Mutation-based confirmation is highly recommended for the inconclusive cases with conventional carrier screening methods to avoid any faulty detection of thalassemia carriers.

## Background

β-thalassemia, characterized by reduced or absent β-globin chain synthesis, is one of the most common inherited blood disorders in the world and hence a major deterrent to public health. Although widespread, the major at-risk populations are mainly from Mediterranean, Middle East and Southeast Asian countries including Bangladesh [1, 2]. WHO reported that approximately 1.5% of global population are carriers of β-thalassemia and 68,000 children are born each year with various thalassemia syndromes [3]. However, precise and up-to-date data on the frequency of β-thalassemia carriers and patients are missing due to lack of accurate diagnostic approach, limited access to information and absence of national screening program in many of the thalassemia-inflicted countries.

Although thalassemia carriers are usually healthy, the patients with β-thalassemia major and HbE/β-thalassemia require lifelong blood transfusion and iron chelation therapy for survival [4]. Cardiac failure due to iron overload and transfusion-related infections have remained the leading causes of deaths of patients with thalassemia [5, 6]. Still, there is no cure for thalassemia except allogeneic bone marrow transplantation (BMT). Also, BMT therapy is too expensive to afford for majority of the world population and the outcome is often unsatisfactory. Due to lack of regular treatment and optimal health care facilities, most thalassemia patients die before adulthood in developing countries [7]. However, many at-risk populations in Cyprus, Greece, Sardinia, Iran etc. have successfully reduced the births of β-thalassemia children by adopting and implementing various preventive measures including nationwide awareness, carrier screening and prenatal diagnosis combined with genetic counseling [8–11].

Although Bangladesh has gained tremendous success in fulfilling Millennium Development Goal-4 by reducing under-5 mortality, there has been an increasingly rapid transition in the burden of disease from primarily communicable to non-communicable diseases. Thalassemia is the single most inherited monogenic blood disorder in Bangladesh and it causes not only substantial morbidity and deaths but also inflicts emotional and financial burden on the family and society [12]. An estimation using limited prevalence data has shown that roughly 33/10,000 newborns are being born each year with thalassemia in Bangladesh [12]. However, thalassemia patient-care and support facilities are barely available in most public and private hospitals. Moreover, health awareness on thalassemia remains highly inadequate among mass population. So, in addition to adoption of proper patient management strategy, prevention by premarital screening and/or prenatal diagnosis should be a useful approach for reducing the risk of thalassemia [7–9, 11]. However, considering socio-religious and financial issues as well as health risk associated with prenatal diagnosis and abortion of the affected fetus, pre-marital screening followed by genetic counseling is arguably the best approach to prevent thalassemia in Bangladesh. In order to weigh the future situation and implement an appropriate policy to tackle thalassemia, precise and up-to-date information on current carrier status is required. The last thalassemia carrier screening was conducted in 2005 on 735 school-going children in Bangladesh [13]. Moreover, the hematological and biochemical methods which are commonly used for screening purposes have limitations as these often end up in false positive and false negative results [14, 15]. In this regard, supplemental molecular methods have been in wide use for their accuracy in carrier screening and predicting severity of the thalassemia patients including their treatment, prognosis and overall management [4, 11]. Recently, the Government of Bangladesh has launched a National Thalassemia Prevention Program. As a part of that strategy, this study was conducted using funds from Non Communicable Disease Control (NCDC) Program, Director General of Health Services, MOHFW, Government of Bangladesh and Rotary Club of Dhaka North. In addition to conventional methods, the study applied DNA-based approaches to determine the accurate status of carriers and also incidence of the at-risk cases with both mutant alleles of HBB gene, which might be responsible for transition from asymptomatic to symptomatic non-transfusion dependent thalassemia in future. Accordingly, the study was conducted on a total of 1877 marriageable-aged participants. Also, a methodical approach was used to accurately determine the division-wise distribution of thalassemia carriers across Bangladesh.

## Methodology

### Study population

This cross-sectional study enrolled a total of 1877 participants (aged between18–35 years) from March 2018 to February 2019 from 10 different (4 universities, 4 medical colleges, and 2 business organization) institutions of Dhaka city with the intent to conduct awareness campaign and screen young unmarried adults. In addition, these institutes are attended by the students and employees from all 8 administrative divisions across Bangladesh. Upon obtaining written informed consent, approximately 5.0 mL of venous blood was collected from each participant via standard venipuncture in EDTA tube. The study was ethically approved by Bangladesh Medical Research Council (BMRC). After completion of the thalassemia screening tests, each participant received the report through email. Those who were found to be carriers of mutations in β- globin gene, were further given an opportunity to receive additional information and counseling.

### Sample size calculation

Total sample size was calculated using the following formula and division-wise sample size was calculated in proportion to national and division-wise population size according to Bangladesh Bureau of Statistics [16].

$$ \mathrm{n}=\frac{{\left(\upalpha +\upbeta \right)}^2\left\{{\mathrm{p}}_1\left(1-{\mathrm{p}}_1\right)+{\mathrm{p}}_2\left(1-{\mathrm{p}}_2\right)\right\}}{{\left({\mathrm{p}}_2-{\mathrm{p}}_1\right)}^2}\times \mathrm{design}\kern0.17em \mathrm{effect} $$

Where, α = the probability of type I error

β = the probability of type II error (power of the test)

p_1_ = 10.2% previously estimated prevalence [13]

p_2_ = 15% expected prevalence

Considering 95% confidence interval (CI), power 80% and a design effect of 2.5, the estimated sample size was 1875. To eliminate bias and for proper representation of each administrative division, samples were collected proportionately to the population size of that division by quota sampling method.

### Analysis of hematological parameters

About 2.0 ml of collected whole blood were used for CBC (Complete Blood Count) analysis to determine RBC indices including hematocrit, mean corpuscular volume (MCV), mean corpuscular hemoglobin (MCH), mean corpuscular hemoglobin concentration (MCHC) and Red cell distribution width (RDW) using automated Hematology analyzer (Sysmex kx-21, Sysmex Corporation, Kobe, Japan).

### Hemoglobin electrophoresis

Hemoglobin electrophoresis was performed on Sebia CAPILLARYS-2 Flex Piercing (Sebia, Lisses, France) using Capillarys Hemoglobin (E) kit to measure HbA, HbA2, HbF and other abnormal Hb variants following manufacturer’s instructions.

### Serum ferritin assay

Serum ferritin was assayed on miniVIDAS® Immunoassay Analyzer (bioMérieux, USA) using VIDAS® FERRITIN kit (bioMérieux SA, Marcy, France) following manufacturer’s instructions.

### Molecular analysis

#### Real-time PCR-based high resolution melting (HRM) curve analysis

Genomic DNA was extracted from whole blood using QIAGEN flexigene® DNA kit (Qiagen, Hilden, Germany) according to manufacturer’s guidelines. Real-time PCR and HRM curve analysis using Precision Melt Analysis™ Software (BioRad) were performed on Bio-Rad CFX96 Real-Time System. This HRM method was previously developed for mutation screening in the β-globin gene of Bangladeshi and regional population of thalassemic endemic countries [2, 17, 18]. This high throughput approach enables to screen mutation(s) in unknown specimens in the presence of reference samples without nucleotide sequencing as well as to screen a large number of samples in a quick and cost-effective manner.

#### Sanger DNA sequencing

In this study, once the mutation positive specimens with a new HRM patterns other than the references were identified, nucleotide sequencing was carried out for those samples to identify the mutation. Sanger DNA sequencing using ABI PRISM-310 software version 3.1.0 (Applied Biosystems) was performed following the polymerase chain reactions (PCR) targeting the mutational hot-spot region of HBB gene for Bangladesh (exon1, intron 1 and a portion of exon 2 of beta-globin gene) and the PCR products purification using the MinElute® PCR purification kit (Qiagen) following the manufacturer’s instructions. Then the retrieved sequence results were compared with the reference sequences (NC_000011.10) for confirmation of the mutation.

### Statistical analysis

The comparison of sensitivity and specificity between traditional methods and molecular approach were performed using https://www.openepi.com/DiagnosticTest/DiagnosticTest.htm with 95% CI. The CI for an observed proportion was calculated using Stata software (version 14.2). With the known genotype frequency, the number of expected newborns with thalassemia was calculated by Hardy-Weinberg equation [19].

## Results

Of the 1877 participants, male to female ratio was 1.5:1 and their average age was 23.4 ± 5.02 (mean ± SD) years (Table 1). The participants were from both rural and urban origins. About 4.32% of the participants had consanguineous parents. Moreover, although all the participants had general education, only 68.14% of them knew the term ‘thalassemia’, whereas 62.3% had no prior knowledge of the disease etiology, severity and risk factors etc. before attending the awareness program, which was arranged as a part of this study.

**Table 1:** Participants’ information regarding gender, parental consanguinity, residence and their knowledge on thalassemia

Characteristic	Parameters	No. of Participants, *n* (%)
Gender	Male	1138 (60.1)
	Female	739 (39.9)
Consanguineous parents	Yes	81 (4.32)
	No	1796 (95.68)
Residence	Urban	1268 (67.6)
	Rural	609 (32.4)
Knowledge regarding thalassemia	Prior knowledge about thalassemia	1279 (68.14)
	Knowledge about how thalassemia is acquired	707 (37.66)
Presence of patients or carriers in the participants family	Yes	50 (2.66)
	No	459 (24.45)
	Not known	1368 (72.88)

### Screening for thalassemia carriers based on MCV, MCH and hemoglobin electrophoresis

The red blood cell count (RBC) and the hematological indices are important in the diagnosis of asymptomatic carriers as almost all kinds of thalassemia carriers show microcytic hypochromic parameters with apparently normal hemoglobin level. Mean corpuscular volume (MCV) and mean corpuscular hemoglobin (MCH) are the two most widely used RBC indices for detecting microcytic hypochromic anemia. In the present study, MCV value of less than 80 fL and/or MCH of less than 27 pg were used as cutoff levels to initially suspect the participants as thalassemia carriers as these are the widely recommended RBC indices for the preliminary screening [20]. Based on these cutoff levels, the study participants (*n* = 1877) were divided into four categories, namely category A, category B, category C and category D. The category A participants had apparently normal RBC indices having MCV greater than or equal to 80 fL and MCH greater than or equal to 27 pg and they constituted 53% (995 out of 1877) of the study samples. Then 612 participants (32.6% of total samples) having MCV and MCH values less than cutoff ranges (< 80 fL and < 27 pg, respectively) had been suspected to have microcytic hypochromic phenotype and were categorized as B. A total of 13 samples with mixed criteria having MCV value less than 80 fL but MCH higher than the cutoff (> 27 pg) were categorized as group C and the remaining samples (257 out of 1877, 15.8%) which had normal MCV (> 80 fL) but MCH less than 27 pg were categorized as D (Table 2).

**Table 2:** Hemoglobin electrophoresis information of the study participants categorized based on MCV and MCH parameters

Groups	No. of participants, n (%) Total, *N* = 1877	Hb electrophoresis results					
		BTT suspects, n (%) (HbA2 > 3.5%)	ETT Suspects, n (%) (HbE = 25–30%)	HbE disease, n (%) (HbE > 90%HbA = 0%)	Others Hb Variants, n (%)	Total participants with abnormal Hb-electrophoresis, n (%)	Normal Hb electrophoresis results, n (%)
Group A (MCV ≥ 80 fL and MCH ≥ 27 pg)	995 (53.0)	3 (0.3)	2 (0.2)	0	4 (0.4)	9 (0.9)	986 (99.1)
Group B (MCV < 80 fL and MCH < 27 pg)	612 (32.6)	41 (6.6)	161 (26.3)	8 (1.3)	5 (0.8)	215 (35.0)	397 (65.0)
Group C (MCV < 80 fL and MCH ≥ 27 pg)	13 (0.7)	0	0	0	0	0	13 (100.0)
Group D (MCV ≥ 80 fL and MCH < 27 pg)	257 (13.7)	2 (0.8)	2 (0.8)	0	0	4 (1.6)	253 (98.4)

*MCV* Mean corpuscular volume, *MCH* mean corpuscular hemoglobin, *BTT* β-thalassemia traits, *ETT, HbE traits* HbE disease refers to the homozygous states of HbE, whereas non-carrier status is defined as “Normal”

Second to MCV and MCH, hemoglobin electrophoresis using Sebia capillary electrophoresis was performed for all the samples as it is the gold standard for thalassemia carrier detections. HbA2 level of > 3.5% was used as a cutoff for screening of β-thalassemia carriers [21, 22]. Since Sebia capillary electrophoresis was able to separate HbA2 distinctly from HbE and other Hb variants like HbD, HbC, HbS and Hb Barts or HbH, the presence of HbE fraction or other hemoglobin variants could indicate the carriers of respective hemoglobin gene mutation.

Table 2 summarizes the results of hematological and electrophoresis analysis of the study participants. About 35% (215/612) of Group B participants had abnormal Hb electrophoresis results compare to 0.9% (9/995), 0% (0/13) and 1.6% (4/257) participants of Group A, C and D, respectively, with abnormal Hb electrophoresis results.

However, in Group-A having apparently normal RBC indices, there were 3 participants with BTT, 2 with ETT and 4 with other Hb variants. In addition, there were two BTT and two ETT carriers among Group-D participants. Finally, all the suspected cases based on MCV, MCH and Hb electrophoresis were subjected to DNA analysis for β-globin gene mutation.

### Second-tier tests using high resolution melt curve analysis and sanger DNA sequencing for detection of β-globin gene mutations

β-thalassemia carriers have generally mild anemia, low MCV and MCH and elevated HbA2 levels. However, there may be considerable variability in hematological phenotype resulting from coexistence with iron deficiency anemia (IDA) and/or coinheritance with alpha thalassemia or delta-globin gene mutations, and presence of silent mutations in HBB gene. These individuals may have milder hematological findings with minimal abnormalities in Hb, MCV, MCH, and HbA2 which may confound the correct diagnosis of β-thalassemia carriers [14]. Considering these facts, a total of 89 samples (Group 1 plus 2 in Table 3) along with samples of BTT, ETT and HbE diseases which had been detected by Hb electrophoresis (Group 3, 4 and 5 in Table 3) were subjected to molecular analysis using HRM curve analysis followed by DNA sequencing. Molecular analysis aimed to (1) avoid faulty detection and confirm that the suspected cases were not left undetected, (2) determine the mutational spectrum of all β-thalassemia and HbE carriers and (3) identify any participants with non-transfusion dependent thalassemia (NTDT). The findings of molecular analysis have been summarized in Table 3.

**Table 3:** Molecular analysis of the selected participants for confirmation of the carrier status

Groups	Selection parameters	Total, n	Mutation Absent, n	Number of participants having mutation in β-globin gene, n	
				Heterozygous	Homozygous^a^/compound heterozygous^b^
Group 1	HbA2 < 2.2% Hb < 10 g/dl	64	64	0	0
Group 2	Hb A2: 3.3–3.5% (borderline suspected)	25	24	01	0
Group 3	HbA2 > 3.5%	46	05	41	0
Group 4	HbE: 25–40%	165	0	163	2^b^
Group 5	HbE > 90% HbA = 0%	08	0	0	8^a^

*Hb* Hemoglobin; ^a^ indicates the presence of two mutations in homozygous condition and ^b^ indicates the presence of compound heterozygous mutation

For Group 1 samples, serum ferritin was measured to confirm IDA and HRM was then performed to detect any coexistence of β-globin gene mutation with IDA. Absence of β-globin gene mutation confirmed that there was no carrier in this group and thus none of the β-thalassemia traits was overlooked because of low level of HbA2.

**Fig. 1:**
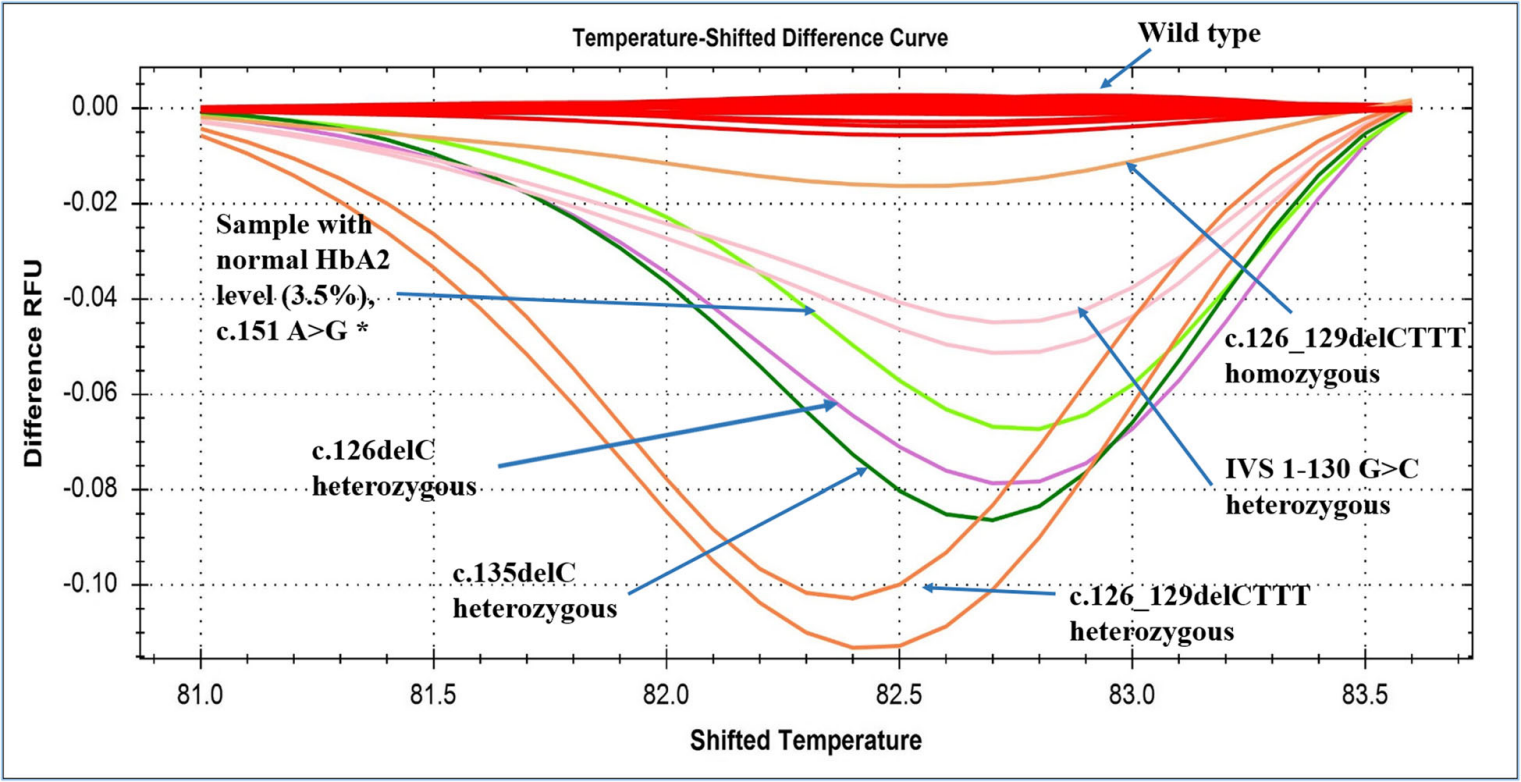
HRM curve analysis for mutation detection in β-globin gene targeting the hot-spot region. The temperature shifted difference curves generated by the mutant alleles of unknown samples could be easily distinguished from the wild type samples and identified by comparing to the controls with known mutations based on differences in the melting curve shapes. RFU, Relative Fluorescence Unit; * indicates novel mutation

From the borderline suspected Group 2, a participant with 3.5% HbA2 generated a HRM curve pattern different from the wild type cluster (without mutation in HBB gene) and also did not match with any of the HRM curves previously established for all the reported mutations in Bangladesh [2]. Sanger sequencing identified and confirmed the suspected mutation as c.151A > G (ACT>GCT; Thr > Ala) in the HBB gene and upon BLAST with databases it was found to be a novel mutation, thereby confirming the carrier status of this participant (Fig. 1).

Identification of a single β-globin gene mutation in 41 out of 46 participants of group-3 could confirm their carrier status. However, of the rest five samples, three having 3.7% HbA2, one with 4% and one having 3.6% HbA2 turned out to be normal in HRM curve analysis and the HRM results were further confirmed by Sanger sequencing. All of these five participants had lower MCH values than the cutoff value (< 27 pg) and three of them showed MCV within normal range i.e. higher than the cutoff value (> 80 fL). Table S1 (Additional file 1: Table S1) shows the hematological features of these 5 participants having HbA2 > 3.5% without mutation in HBB gene.

Molecular analysis was also able to identify 2 participants (out of 165 HbE carriers based on Hb electrophoresis) with compound heterozygous mutations (c.79G > A + c.92 + 5G > C); one in Hb-E allele and another one in the trans-allele to HbE. These participants had relatively high levels of fetal hemoglobin (HbF of 2.8 and 11.4%), which also could justify the presence of mutations in both alleles because presence of a mutation in the allele which is trans to HbE allele (c.79G > A mutation) induces higher levels of HbF production. Hemoglobin indices of the two samples have been summarized in Table 4. However, homozygous c.79G > A was identified in all 8 participants of Group 5, thus confirming their HbE disease status. Therefore, the study identified a total of 163 HbE carriers, 42 β-thalassemia carriers and 8 participants with HbE disease.

**Table 4:** Hemoglobin indices of the two participants containing compound heterozygous mutation

Participant No.	Age (Years)	Hb (g/dl)	MCV (fL)	MCH (pg)	RDW (%)	HbA (%)	HbF (%)	HbE (%)	HbA2 (%)
1	20	9.7	69.6	20.8	19	63.5	11.4	22.1	3
2	21	9.9	66	19	19.3	79.3	2.8	14.3	3.6

Next we wanted to compare the sensitivity and specificity between traditional hematological analysis and HRM-based molecular approach (Table 5). Compared to the molecular method with 100% sensitivity and 100% specificity, combination of CBC and Hb electrophoresis tests showed 99.55 (95% CI, 97.51–99.92) sensitivity and 99.82 (95% CI, 99.47–99.94) specificity. Furthermore, only Hb electrophoresis gave 5 false positive and one false negative results, whereas the combination of CBC and Hb electrophoresis resulted in faulty detection in 4 cases.

**Table 5:** Comparison of sensitivity and specificity among the conventional hematological-analysis based approaches for thalassemia carrier detection

Screening method	Mean sensitivity, % (95% CI)	Mean specificity, % (95% CI)	Positive predictive value, % (95% CI)	Negative predictive value,% (95% CI)	Diagnostic accuracy, % (95% CI)	True +ve (n)	False + ve (n)	True –ve (n)	False -ve(n)
MCV + MCH only	96.04 (92.64–97.9)	59.76 (57.37–62.17)	24.72 (21.98–27.67)	99.1 (98.29–99.52)	64.14 (61.95–66.28)	218	664	986	9
Hb electrophoresis	99.55 (97.51–99.92)	99.7 (99.29–99.87)	97.81 (94.97–99.06)	99.94 (99.66–99.99)	99.68 (99.3–99.85)	223	05	1648	01
MCV+ MCH and Hb Electrophoresis	99.55 (97.51–99.92)	99.82 (99.47–99.94)	98.67 (96.17–99.55)	99.94 (99.52–99.96)	99.79 (99.66–99.99)	223	03	1650	01

*CI* Confidence Interval, *MCV* Mean corpuscular volume, *MCH* Mean corpuscular hemoglobin

### Mutation spectrum in the HBB gene of the study participants with thalassemia traits

As shown in Table 6, out of nine different mutations including a novel mutation, the most common mutation was c.79G > A (CD 26/ HbE) (73.42%) followed by c.92 + 5G > C (14.41%).

**Table 6:** Mutation spectrum of β-globin gene in the β-thalassemia and HbE carriers in Bangladeshi population

SL No.	Mutation Pattern	Number of samples, n (%; 95% CI)
1	c.79G > A (HbE)	163 (73.42; 67.62–79.22)
2	c.92 + 5G > C	32 (14.41; 9.8–19.02)
3	c.79G > A + c.79G > A	8 (3.61; 1.16–6.06)
4	c.92 + 130G > C	01 (0.45; 0.43–1.33)
5	c.151A > G ^a^	01 (0.45; 0.43–1.33)
6	c.126_129delCTTT	03 (1.35; 0.16–2.86)
7	c.27_28insG	02 (0.90; 0.34–2.14)
8	c.47G > A	03 (1.35; 0.16–2.86)
9	c.79G > A + c.92 + 5G > C	02 (0.90; 0.34–2.14)

^a^novel mutation; not reported in Bangladeshi population and also globally [23]

### Frequency of thalassemia carriers and other hemoglobinopathies among Bangladeshi population

ETT was found to be the most prevalent trait with a frequency of 8.68% (163/1877) followed by 2.24% BTT (42/1877) and; thus ETT and BTT together comprised of a total frequency of 10.92% (205/1877). Moreover, participants with HbD trait, asymptomatic HbE disease, suspected NTD HbE-β-thalassemia, hereditary persistence of fetal hemoglobin (HPFH) and α-thalassemia trait were also identified (Table 7). Altogether, 11.89% (223/1877) participants were carriers of abnormal hemoglobin genes.

**Table 7:** Distribution of thalassemia carriers and other Hb variants among the study participants

Types of thalassemia carriers and other Hb variants	Number of participants, n	Frequency %, (95% CI)
HbE Trait (ETT)	163	8.68 (7.41–9.95)
β-thalassemia trait (BTT)	42	2.24 (1.57–2.91)
HbE disease	08	0.43 (0.13–0.73)
α-thalassemia trait	02	0.11 (0.04–0.26)
HbD trait	03	0.16 (0.02–0.34)
Suspected NTD HbE/β-thalassemia	02	0.11 (0.04–0.26)
HPFH	03	0.16 (0.02–0.34)
Total carriers of mutations in one or both alleles of globin genes	223	11.89 (10.43–13.35)

*CI* Confidence Interval, *Hb* Hemoglobin, *HPFH* Hereditary persistence of fetal hemoglobin, *NTD* Non-transfusion dependent

### Contribution of consanguinity to increase thalassemia carrier frequency in the country

There were 81 (4.32%; 95% CI, 3.4–5.24) participants of consanguineous parents. The carrier frequency among the participants with history of consanguinity was 23.5% (19/81), whereas it was almost half (11.4%, 204/1796) among the children of non-consanguineous parents. The highest consanguinity was observed in Rangpur division (8/140; 5.7%), which also had the highest carrier frequency among the eight administrative divisions of Bangladesh. The findings suggest that consanguinity contributes significantly to the increased rate of thalassemia in Bangladesh.

### Distribution of β-thalassemia and HbE carriers across eight divisions of Bangladesh

**Fig. 2:**
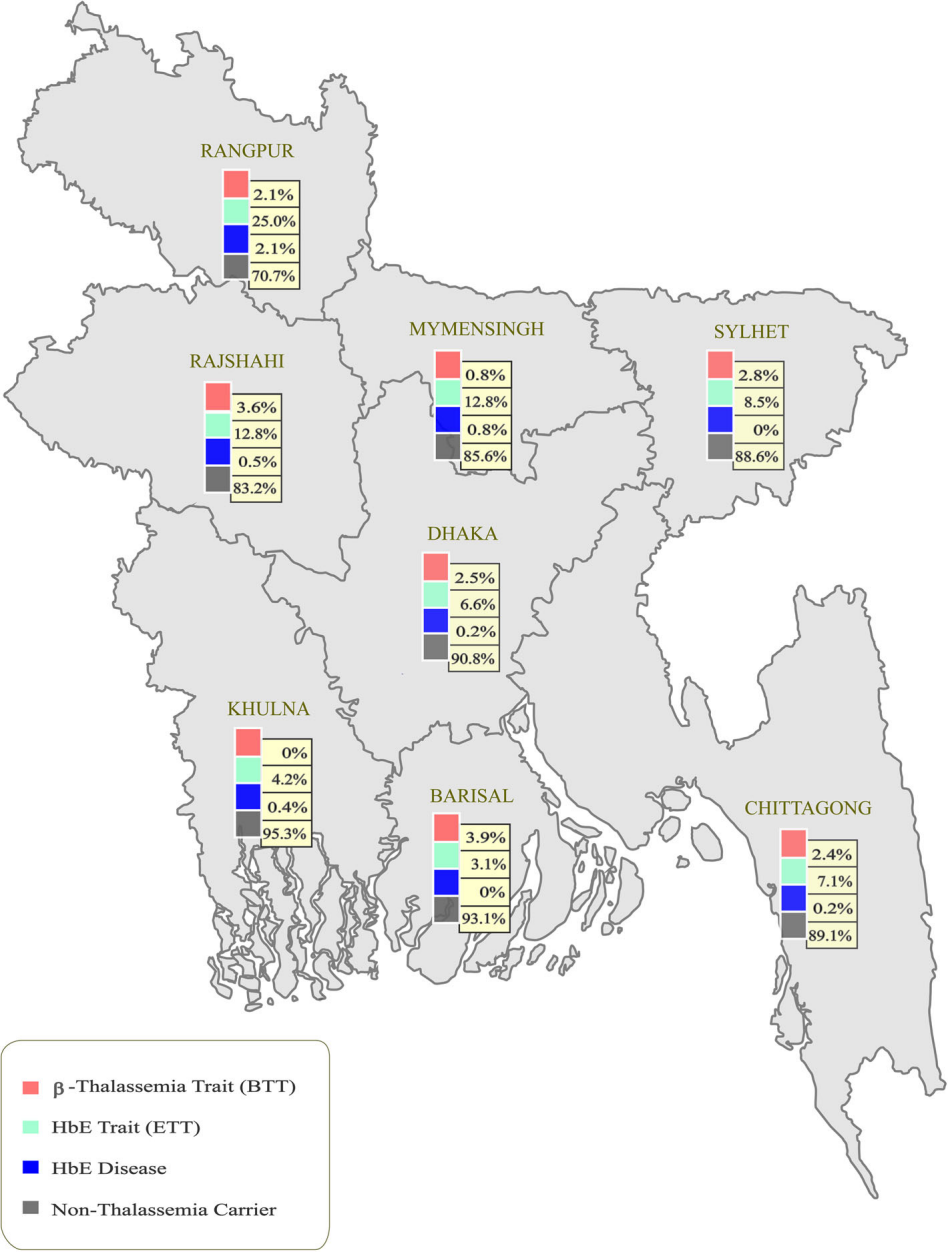
Map showing the frequency of β-thalassemia and HbE carriers across eight administrative divisions of Bangladesh

We found that the frequency of ETT was higher than that of the BTT across all divisions except Barisal (Fig. 2). The ETT frequency varied from as low as 4.2% (95% CI, 1.65–6.75) in Khulna Division to as high as 25% (95% CI, 17.83–32.17) in Rangpur. Conversely, the highest BTT frequency was found in Barisal Division (3.9%; 95% CI, 0.57–7.23). Unexpectedly, we could not detect any participants with BTT in Khulna Division. The highest frequency of BTT plus ETT was found in Rangpur division (27.1%; 95% CI, 19.74–34.46) followed by Rajshahi Division (16.4%; 95% CI, 11.22–21.58).

## Discussion

This is the first thalassemia carrier screening study conducted among young individuals of marriageable-age in Bangladesh. The study aimed to determine (1) nationwide carrier frequencies of β-thalassemia and HbE traits more precisely using molecular approach as supplementation to hematological and electrophoretic indices for rectification of the false positive or false negative cases (2) distribution of division-wise carrier frequencies, and (3) the mutation spectrum in the HBB gene of the carriers.

Carrier frequency of ETT plus BTT was 10.92% (95% CI, 9.51–12.33), where ETT had the highest frequency (8.68%; 95% CI, 7.41–9.95) followed by BTT (2.24%; 95% CI, 1.57–2.91). A previous study conducted in 2005 by Khan et al. reported an average frequency of 10.2% in Bangladeshi population with 6.1% ETT and 4.1% BTT [13]. This difference in frequencies between these two studies can be explained by the fact that previous studies used only conventional hematological approaches which often give false positive and false negative results [14, 15], whereas the present study applied molecular approach to avoid faulty detection of any carriers. Moreover, this study was conducted on a larger number of participants and the number of total participants as well as division-wise enrollment was enumerated statistically in proportion to the population size of each division for a precise apprehension of distribution of carrier frequency. Other studies conducted in neighboring India and Sri Lanka reported the comparable BTT frequencies of 2.68–4.05% and 2·2%, respectively, where the ETT frequencies were 3.4 and 0.5%, respectively [24–26]. However, our study showing an alarmingly high ETT frequency (8.68%) is supported by the previous study in Bangladesh [13]. Among eight administrative divisions, the highest carrier frequency was detected in Rangpur Division, where the HbE carrier frequency was 25%. Previous study showed the highest thalassemia carrier frequency in Rajshahi Division because the newly created administrative region of Rangpur was the part of Rajshahi Division during that study.

The first line of screening for identification of thalassemia carriers is a complete blood count followed by a measurement of HbA2 and HbF proportions. Since screening by hematological indices may result in misdetection of carriers due to factors like co-inheritance of α-thalassemia, mutations in β- and δ-globin genes, mutations in KLF1 gene and iron deficiency anemia [15, 27, 28], this study applied a comparatively cheaper and easy-to-perform DNA-based HRM curve analysis method to confirm and characterize mutations in the β-globin gene. This method supplements the conventional hematological and electrophoretic parameter based approaches for identification of the thalassemia carriers and patients [2]. For example, although the parameters MCV ≥ 80 fL and MCH ≥ 27 pg are usually considered as a negative indicator for HbE trait, our study using electrophoresis had identified two HbE carriers with MCV ≥ 80 fL and MCH ≥ 27 pg and a case having MCV > 80 fL and MCH < 27 pg as HbE carrier, indicating the shortcomings of hematological indices in screening of HbE carriers. Similarly, five participants with HbA2 > 3.5%, which is a widely used indicator of beta thalassemia trait, turned out to be normal by HRM curve analysis and Sanger sequencing, thereby further demonstrating shortfalls of Hb electrophoresis in detection of thalassemia carriers. The higher HbA2 might be caused by mutations in the KLF1 gene leading to borderline high HbA2 and thus may result in false positive findings in Hb electrophoresis [15]. Moreover, a number of studies reported frequent detections of high HbA2 levels in healthy individuals without any mutation in the HBB gene [29, 30]. On the other hand, specimens with HbA2 level in the borderline range (3.3–3.5%) with low MCV and/or low MCH must be subjected to DNA-based analysis to determine the carrier status of the participants. Notably, borderline HbA2 level might result from coinheritance of β-globin gene mutations with iron deficiency anemia and α thalassemia traits that usually lower the level of HbA2 to normal or borderline range in the β-thalassemia carriers [31]. In this study, all the specimens with abnormal hematological indices were tested using high resolution melt (HRM) curve analysis to confirm the presence of mutation in β-globin gene and if the molecular tests were not performed, about 5 in every 1000 carriers of the β-thalassemia and HbE variants would have been missed and around 1.8% cases could have been interpreted erroneously. Therefore, although the combination of MCV, MCH and Hb electrophoresis resulted in high sensitivity and specificity, the DNA-based approaches like HRM curve analysis and Sanger sequencing had been proved to be very useful to avoid false positive and false negative results by detecting mutations in the β-globin gene, and thereby confirming the true thalassemia carrier status of the participants. In a previous study, we described the advantages and cost-effective nature of this approach over other DNA-based screening methods like Denaturing High Performance Liquid Chromatography (DHPLC), Single Strand Conformational Polymorphism (SSCP) and Denaturing Gradient Gel Electrophoresis (DGGE), Amplification Refractory Mutation System (ARMS) PCR, Sanger nucleotide sequencing etc. [2]. Notably, this real time PCR-based high throughput HRM curve analysis is easy to perform and time-saving as there is no need of post PCR amplification processes like PCR product purification, gel electrophoresis etc. All these advantages offered by the HRM-based techniques make it an ideal candidate for molecular screening of thalassemia in countries of thalassemia belt and resource limitation.

More importantly, this study identified two clinically asymptomatic individuals with pathogenic mutations in both alleles of HBB gene using HRM method, who were detected as carriers by Hb electrophoresis. Although the association between genotype and phenotype is established for both α- and β-thalassemia, differentiation into various phenotypes of thalassemia is mostly based on clinical signs and symptoms. However, the same mutations i.e. c.79 G > A + c.92 + 5G > C have been reported as pathogenic in patients with mild to severe form of HbE-beta thalassemia [32, 33]. Hence, although currently these two individuals who seem to be carriers without any clinical manifestations, they might be at risk of developing non-transfusion dependent thalassemia (NTDT) in future. In our ongoing study on the role of various genetic modifiers on clinical heterogeneity of thalassemia patients in Bangladesh, the age of first transfusion of NTDT patients ranged from 13 to 60 years (unpublished data). It should also be mentioned that patients with hemoglobin E/β-thalassemia show different phenotypic variability at different stages of development [34]. Since numerous factors have been identified to be associated with disease severity of the NTDT patients, identification of NTDT patients is vital for prognosis because increased intestinal iron absorption in such patients increases the risk of thrombotic disease, pulmonary hypertension, sudden cardiac arrest, and liver damages etc. [34–36]. Thus the significance of diagnosis of NTDT is crucial because a timely treatment intervention will curtail the progression of disease severity and thus prevent an untimely death. All these aspects emphasize on the requirement of molecular-based carrier screening which is the ultimate confirmation of a carrier status. Furthermore, the study identified 9 different mutations including a novel mutation (c.151A > G) in the beta-globin gene of the carriers. Further studies are needed to know the pathogenesis of this novel mutation.

With the current ETT plus BTT carrier frequency of 10.92%, 9176 babies are born with thalassemia each year (according to Hardy-Weinberg equation), thereby further worsening the situations of thalassemia patients [13, 19]. Moreover, the study found that the thalassemia carrier frequency was almost double among the children with history of consanguineous marriage, a common socio-culture of this region and thus consanguinity contributes to increased burden of thalassemia. Even though majority of the participants were university/college going students, 62% of them did not know the disease etiology and about 32% did not have any knowledge about the disease prior to enrolment suggesting that the knowledge regarding thalassemia is quite insufficient among the mass population.

At present, the yearly medical cost required for thalassemia patient ranges from $1632 to $3960 in Bangladesh and there is neither a national insurance facility nor a subsidized or free treatment system from the government [12], suggesting a severe health, economic and emotional burden to the nation and thus adoption of a national thalassemia prevention strategy is a demand of time. Several thalassemia endemic countries have set up comprehensive national prevention programs, which include public awareness and education; carrier screening using molecular diagnostics, genetic counseling and prenatal diagnosis [37, 38]. Effectiveness of such prevention program in Sardinia is evidenced by a reduction in the birth rate with thalassemia major from 1:250 live births to 1:4000 and such success is also achieved by other countries including Cyprus, Iran, and Turkey [10, 38, 39]. Although carrier screening and counseling are being performed on a voluntary basis in some countries, countries like UAE, Saudi Arabia, Jordan, Cyprus, Iran and Turkey are performing compulsory premarital screening for thalassemia to discourage marriage between two carriers [38]. These strategies may help guide health policy makers of Bangladesh to adopt an appropriate thalassemia prevention strategy considering the available resources, religious values and social culture.

In summary, as prevention of thalassemia is far cheaper and better than treatment and currently no affordable cure is available, an immediate and concerted action on thalassemia prevention should be made mandatory in Bangladesh. A massive awareness program targeting general population and an intensive educational program for health personnel including physicians, nurses, health and family planning workers should be carried out nationwide promptly. Also, appropriate screening methods combining hematological, electrophoretic and molecular approaches associated with genetic counseling should be required in existing hospitals and health facilities. The information of this study will be helpful in several ways, such as measuring nationwide carrier frequency with accuracy and grasp the gravity of the situation, identifying the at-risk population and thus prioritizing them, and necessities and benefits of molecular-based carrier screening. Moreover, this study demonstrated the feasibility and usefulness of cost-effective HRM approach in resource limited settings which can be followed in other countries of thalassemia-belt for detection of HBB gene mutations and confirmation of the carrier status.

## Conclusion

This study highlights that adoption of a molecular screening method for detection of mutations in the HBB gene could overcome the shortcomings of the conventional methods, in particular, for prenatal and newborn screening and for confirmation of the inconclusive cases by the traditional approaches. With the current carrier frequency, HbE/β-thalassemia will be posing a tremendous threat to the public health of Bangladesh if necessary measures like awareness program for mass population and medical personnel and; establishment of carrier screening facilities aligned with genetic counselling in health centers and hospitals across the country, are not implemented immediately. Lastly, the HRM-based cost-effective molecular methods can be initiated in other thalassemia-prone countries and help in fighting these non-curable and life-threatening disorders.

## Supplementary information

**Additional file 1: Table S1.** Hematological features of the participants having high HbA2 level (HbA2 > 3.5%) without mutation in beta globin gene of hemoglobin.

## Data Availability

All relevant data are within the paper. Further information is available from the authors on request.

## Abbreviations

BMT: Bone Marrow Transplantation
BTT: Beta-thalassemia trait
CBC: Complete Blood Count
CI: Confidence Interval
EDTA: Ethylenediamine tetra acetic acid
ETT: HbE Trait
fl: Femtolitre
Hb: Hemoglobin
HBB: Hemoglobin beta subunit gene
HPFH: Hereditary Persistence of Fetal Hemoglobin
HRM: High Resolution Melting
IDA: Iron Deficiency Anemia
KLF1: Krueppel-like factor 1
MCH: Mean Corpuscular Hemoglobin
MCV: Mean Corpuscular Volume
NTDT: Non Transfusion Dependent Thalassemia
PCR: Polymerase Chain Reaction
pg: Pictogram
WHO: World Health Organization
