# Supplementary material for: Nationwide carrier detection and molecular characterization of β-thalassemia and hemoglobin E variants in Bangladeshi population
Source: Orphanet J Rare Dis. 2020 Jan 15;15:15. doi: 10.1186/s13023-020-1294-z (PMC6961315; doi:10.1186/s13023-020-1294-z)
Supplement: Supplementary file 1 — Additional file 1: Table S1. Hematological features of the participants having high HbA2 level (HbA2 > 3.5%) without mutation in beta globin gene of hemoglobin. [file 13023_2020_1294_MOESM1_ESM.docx]

| **Age**  **(years)** | **Gender** | **HbA2 (%)** | **HbF (%)** | **RBC count (X10^6^/µl)** | **Hb level (g/dL)** | **HCT (%)** | **MCV (fL)** | **MCH (pg)** | **MCHC (g/dL)** | **RDW (%)** |
| --- | --- | --- | --- | --- | --- | --- | --- | --- | --- | --- |
| 21 | Male | 3.7 | 0 | 5.18 | 13.6 | 41.6 | 80.3 | 26.3 | 32.7 | 12.5 |
| 21 | Male | 3.7 | 0 | 5.34 | 13.3 | 41.6 | 77.9 | 24.9 | 32.0 | 12.9 |
| 23 | Male | 4.0 | 0.2 | 5.8 | 15.4 | 46.9 | 80.9 | 26.6 | 32.8 | 13.9 |
| 21 | Male | 3.7 | 0 | 6.79 | 15.5 | 48.3 | 71.1 | 22.8 | 32.1 | 15.4 |
| 24 | Female | 3.6 | 0 | 4.92 | 13.3 | 40.5 | 82.3 | 27 | 32.8 | 12.6 |
| ** Hb, hemoglobin; MCV, mean corpuscular volume; MCH, mean corpuscular hemoglobin; HCT, hematocrit; MCHC, mean corpuscular hemoglobin concentration; RDW, red cell distribution width. | | | | | | | | | | |

Additional file 1: **Table S1**: Hematological features of the participants having high HbA2 level (HbA2> 3.5%) without mutation in beta globin gene of hemoglobin.
